# Preparation, characterization, and evaluation (*in-vitro*, *ex-vivo*, and *in-vivo*) of naturosomal nanocarriers for enhanced delivery and therapeutic efficacy of hesperetin

**DOI:** 10.1371/journal.pone.0274916

**Published:** 2022-11-03

**Authors:** Shailendra Gurav, Poonam Usapkar, Nilambari Gurav, Sameer Nadaf, Muniappan Ayyanar, Rucheera Verekar, Ritesh Bhole, Chintha Venkataramaiah, Goutam Jena, Rupesh Chikhale

**Affiliations:** 1 Department of Pharmacognosy, Goa College of Pharmacy, Panaji, Goa University, Goa, India; 2 PES’s Rajaram and Tarabai Bandekar College of Pharmacy, Ponda, Goa University, Goa, India; 3 Sant Gajanan Maharaj College of Pharmacy, Mahagaon, Chinchewadi, Maharashtra, India; 4 Department of Botany, A. Veeriya Vandayar Memorial Sri Pushpam College (Autonomous), Affiliated to Bharathidasan University, Poondi, Thanjavur, Tamil Nadu, India; 5 Dr. D. Y. Patil Institute of Pharmaceutical Sciences and Research, Pimpri, Pune, Maharashtra, India; 6 Department of Medical Environmental Biology and Tropical Medicine, School of Medicine, Kangwon National University, Chuncheon, Republic of Korea; 7 Roland Institute of Pharmaceutical Sciences, Berhampur, Odisha, India; 8 UCL School of Pharmacy, London, United Kingdom; The Islamia University of Bahawalpur Pakistan, PAKISTAN

## Abstract

The present study intends to formulate, characterize and appraise the phospholipid-based nanovesicular system for enhanced delivery of Hesperetin (HT). The quality by design (QbD) approach was employed to prepare Hesperetin naturosomes (HTN) using the solvent evaporation technique and assessed for physicochemical and pharmacological attributes. The FTIR, DSC, and PXRD studies confirmed the successful formation of a vesicular drug-phospholipid complex, while photomicroscopy, SEM, and TEM analysis revealed the morphology of HTN. The functional attributes substantially enhanced the HT’s aqueous solubility, drug release, and membrane permeation. The aqueous solubility of HTN was ~10-fold more than that of pure HT. Likewise, the *in-vitro* dissolution data of HTN showed better competence in releasing the HT (>93%) than the pure HT (~64%) or the physical mixture (~74%). Furthermore, HTN significantly altered HT permeation (>53%) when compared to pure HT (23%) or the physical mixture (28%). The current study showed that naturosomes are a promising way to improve the solubility in water, bioavailability, and therapeutic effectiveness of drugs.

## Introduction

Citrus fruits are the primary source of Hesperetin (HT), a naturally occurring flavanone class of flavonoids generated from the hydrolysis of hesperidin (hesperetin 7-rhammnoglucoside). CYP1A and CYP1B1 (cytochrome P450 isoforms) selectively metabolize HT to eriodictoyl, signifying O-demethylation of HT in the liver. The primary metabolites of HT are 7-O-glucuronide and 3’-O-glucuronide, which are generally discovered in urine but not in feces, showing that colon bacteria further break down HT to ring fission products and phenolic acids [1,2].

Shorter half-life and rapid clearance limit the use of HT as a potent biomolecule for its various biological activities (anticarcinogenic, antioxidant, antibacterial, antiviral, anti-inflammatory, and hepatoprotective) [3]. Despite many advantages of HT, low water solubility and stability constrain its use and consequently exert low biological activity [4].

Numerous strategies, including the production of emulsions, liposomes, and nanoparticles, as well as the modification of chemical structures and the administration of prodrugs, have been proposed as potential solutions to the problem of poor solubility and bioavailability. Naturesomes have emerged as a promising technique for increasing phytoactives’ bioavailability [5]. Naturosomes are better absorbed and improve bioavailability, dose reduction, and increase the duration of action than conventional herbal drugs or extracts. Moreover, recently, the complexation of plant actives with dietary phospholipids has proven to be a successful strategy for increasing the usage of therapeutically active drugs [6,7]. Hence, HT is a good candidate for the preparation of naturosomes.

A new pharmaceutical and valid form of the flavonoid drug with enhanced therapeutic efficacy could be developed using naturosomal nanocarriers, which have favorable pharmacokinetic properties, superior efficacy, and tolerability profile. Therefore, the current investigation deals with the formulation and evaluation of HT naturosomes (HTN) to upsurge the solubility, bioavailability, and therapeutic efficacy of HT.

## Materials and methods

### Materials

HT was obtained from Sigma Chemicals, Co., St. Louis, USA. The phospholipid, LECIVA-S70, was provided as a gift sample by VAV Life Sciences, India. The other chemicals and reagents used were of the highest analytical grade.

### Preparation of HT Naturosome (HTN)

HTN was prepared by a solvent evaporation technique using the Quality by Design (QbD) approach. Briefly, HT and LECIVA-S70 (PC) were taken in different ratios like 1:0.5, 1:1.11, 1:2, 1:2.89, or 1:3.5, separately dissolved in ethanol (10 mL); the two solutions were mixed and placed in a rotary evaporator. The reaction was maintained at different temperatures like 40, 44, 50, 56, or 60°C using a water bath for different durations, i.e., 60, 84, 120, 156, or 180 min. The obtained clear solution was concentrated to 2–3 mL and treated with an excess of n-hexane to eliminate any traces of solvents. Finally, the HT-phospholipid complex, i.e., HTN, was stored in amber-coloured glass vials [8].

### QbD experimental approach

QbD enables better, faster, and more competent product development and helps to understand the roles of excipients in the formulation and the process by employing a science and risk-based strategy for excipient selection (compatibility and functionality) and better excipient characterization [1]. A central composite design using twenty possible combinations was used to investigate the combined effect of three variables, i.e., drug: phospholipid ratio (X_1_), reaction temperature (X_2_), and reaction time (X_3_), on entrapment efficacy (Y_1_; %) (Tables 1 and 2) [8].

**Table 1:** Coded levels and “Real” values for each factor under study.

Variables	Levels				
	-1.732	-1	0	+1	+1.732
***Independent***	**Real values**				
Drug: Phospholipid ratio (X_1,_ mol: mol)	0.5	1.11	2	2.89	3.5
Reaction temperature (X_2_, °C)	40	44	50	56	60
Reaction time (X_3_, mins)	60	84	120	156	180
***Dependent***					
Entrapment efficiency (Y, % w/w)					

**Table 2:** Central composite design formulation batches with respective entrapment efficiencies.

Batches	X_1_	X_2_	X_3_	Entrapment efficiency* (%)
F1	-1	-1	-1	68.19 ± 0.85
F2	1	-1	-1	79.58 ± 1.21
F3	-1	1	-1	80.11 ± 0.79
F4	1	1	-1	88.56 ± 1.16
F5	-1	-1	1	79.61 ± 1.27
F6	1	-1	1	88.98 ± 0.85
F7	-1	1	1	88.83 ± 1.18
F8	1	1	1	97.65 ± 1.09
F9	-1.732	0	0	74.26 ± 0.88
F10	1.732	0	0	93.77 ± 1.17
F11	0	-1.732	0	75.53 ± 1.28
F12	0	1.732	0	89.49 ± 0.98
F13	0	0	-1.732	81.07 ± 1.05
F14	0	0	1.732	92.41 ± 1.16
F15	0	0	0	86.12 ± 1.21
F16	0	0	0	85.95 ± 1.09
F17	0	0	0	86.03 ± 1.05
F18	0	0	0	84.78 ± 1.28
F19	0	0	0	87.16 ± 0.97
F20	0	0	0	86.39 ± 1.12

The following Eq (1) was used to analyze the response using a statistical model that included interactive and polynomial terms;

$$Y=b_{0}+b_{1}X_{1}+b_{2}X_{2}+b_{3}X_{3}+b_{11}X_{1}{}^{2}+b_{22}X_{2}{}^{2}+b_{33}X_{3}{}^{2}+b_{12}X_{1}X_{2}+b_{23}X_{2}X_{3}+b_{13}X_{1}X_{3}$$ (1)

Where Y is the dependent variable, *b*_*0*_ is the 20-run arithmetic mean response, and *b*_*i*_ is the calculated coefficient for the factor X_i_. The main effects (X_1_, X_2,_ and X_3_) represented the average consequence of increasing the value of one factor at a time from low to high. The interaction terms (X_1_X_2_, X_2_X_3_, and X_1_X_3_) demonstrated altered responses with concurrent variations in all three variables. Finally, the linearity was investigated using the polynomial terms (X_1_^2^, X_2_^2^, and X_3_^2^).

### Entrapment efficiency (EE) of HTN

The entrapment analysis was carried out to calculate drug EE by the indirect method. First, the amount of the drug unentrapped was separated from the complex using a solvent in which the drug is soluble, but the complex is not. Briefly, HTN powder (100 mg) was dispersed in 10 mL of DMSO and kept on a shaker for 30 min at 150 rpm, followed by filtration. Further, the filtrate was subjected to UV-spectrophotometric (Shimadzu, UV-2700) analysis at 287 nm [8,9]. Finally, the EE of the prepared complex, i.e., HTN, was calculated using the following Eq (2):

$$\text{EE}\left(\%\right)={\text{C}}_{\text{t}}-{\text{C}}_{\text{f}}/{\text{C}}_{\text{t}}\;\text{X}\;100$$ (2)

Where C_t_ = Total concentration of HT, C_f_ = HT contained in the filtrate solution. The concentration of HT was estimated using a straight line equation, y = 0.016x + 0.0933 (r^2^ = 0.9965).

### HT content

Using the method described in section 2.4 and Eq (3), the amount of HT in the HTN was measured;

HTcontent=Amt.ofHTintheHTN/Amt.ofHTNX100 (3)

### Preparation of optimized formulation

An optimized batch of HTN was prepared using 0.6 g of HT and 1.734 g of PC following the procedure mentioned in section 2.2 and stored in amber-colored glass vials.

### Physicochemical characterization

#### Fourier transform infrared spectroscopy (FT-IR)

FT-IR analysis of HT, PC, physical mixture (PM) of HT and PC, and HTN was carried out using an FT-IR spectrophotometer (Model: IR Affinity-1S). Test samples were kept below the probe and scanned for 45 scans between 400–500 cm^-1^ [8].

#### Differential scanning calorimetry (DSC)

An aluminum crimp cell with HT, PC, PM, and HTN was used for the DSC investigation and heated at 10°C/min from 0 to 400°C in a nitrogen environment at a flow rate of 50 mL/min. Peak transition onset temperatures were recorded using an analyzer (Shimadzu, DSC-60) [8].

#### Photomicroscopy

An HTN suspension prepared in distilled water was placed on a microscope slide and subjected to photomicroscopy using an optical microscope paired with a camera (Nikon, Eclipse) [8].

#### Scanning electron microscopy (SEM)

Briefly, the HTN was layered on double-sided carbon tape and a brass stub, followed by a thin coat of gold-palladium. Further, the palladium-coated samples were subjected to SEM imaging using a Scanning Electron Microscope (JEOL JSM 5600) equipped with a digital camera (Zeiss, EVO special edition) [8].

#### Transmission electron microscopy (TEM)

In the TEM study, a minor quantity of HTN was negatively stained with 2% uranic acid and placed on a copper grid of a TEM analyzer (Jeol, JEM 2100) [8].

#### Particle size and zeta potential analysis

Photon correlation spectroscopy, with dynamic light scattering on Zetasizer nano (Malvern Nano series S90 Zeta sizer), was used to measure the particle size of HTN. Moreover, Smoluchowski’s equation measured the zeta potential from the electrophoretic mobility of HTN with the help of the same instrument used for particle size analysis [8].

#### X-ray diffraction analysis (XRD)

HTN was subjected to XRD analysis over 5–60° (2θ) using the Cu-Anode X-ray tube and scintillation detector (Rigaku, Ultima IV), following the operating conditions of a voltage of 40 kV; current of 20 mA; scanning speed of 1/min [8–10].

### Functional evaluation

#### Solubility study

The solubility of HT, PC and HTN was determined by adding them to volumetric flasks with 10 mL of different solvents and shaking the contents [8].

#### Apparent solubility

At room temperature, 10 mL of water or n-octanol were mixed with excess HT and HTN in sealed glass containers, followed by 24 h of agitation on a rotary shaker. Further, the supernatant was collected using a centrifuge (4000 rpm, 30 min) and was analyzed for HT using a UV-spectrophotometer using an equation y = 0.016x + 0.0933 (r^2^ = 0.9965) [8].

#### Dissolution study

USP type-II dissolution apparatus was used for the *in-vitro* dissolution studies of HT, HTN, and PM. Briefly, a quantity of HTN (**≈**50 mg of the HT) was added to the agitated dissolution medium (900 mL phosphate buffer, pH 6.8) at 100 rpm and 37°C. Then, 10 mL of samples were periodically extracted and replaced with fresh medium to maintain the sink conditions. Finally, the samples were membrane filtered and analyzed using UV at 287 nm [8].

### *Ex-vivo permeability* study

Wistar albino rat was anaesthetized with a high dosage of thiopental (35 mg/kg, i.v.) and sacrificed via cervical dislocation to get the dorsal side of the rat’s skin for experimental use. A trimmer was used to remove the rat’s hair. The removed skin was cleaned in distilled water and placed on a Franz diffusion cell, with the stratum corneum facing the donor compartment and the dermis facing the receptor compartment. HTN, in a specified amount, was loaded onto the skin in the donor compartment and slightly immersed in 50 mL of receptor medium. A magnetic stirrer was used to agitate the cell content at 37±0.5°C. The reported method analyzed the drug concentration in an aliquot of 5 mL taken at regular intervals up to 8 h. After each withdrawal, the diffusion medium was changed with a fresh volume of the same diffusion medium [8].

### *In-vivo* biological activity

#### Animals

Wistar rats (wt 220–250 g) were kept in groups at room temperature in a 12:12–h light:dark cycle, with unrestricted access to food and water except during the experiment. Animal studies were performed strictly according to the Committee for the Purpose of Control and Supervision of Experiment on Animals (CPCSEA) guidelines and with prior approval (No. GCP/IAEC/2018/14) of the Institutional Animal Ethics Committee (IAEC) of Goa College of Pharmacy, which is registered under CPCSEA, Government of India with the 611/02/C/CPCSEA.

#### Drug preparation

Test drugs, i.e., HT and HTN (50 mg/kg), were administered orally for 21 days, 1 h before footshock, in carboxymethyl cellulose (0.3%) suspensions prepared in distilled water. Furthermore, *Panax ginseng* (PG) root powder (100 mg/kg) was administered as a standard, and the control group was directed only to the vehicle (2.5 mL/kg po).

#### Footshock-induced stress

As per our previously published reports, the stress induction method was adopted [8]. First, an hourly grid floor footshock was used on the rats. It was programmed to deliver two 2-mA shocks every 3–5 s, with a 10 to 110 s interval between each shock. Footshock stress was maintained for 21 days.

#### Elevated plus-maze (EPM) apparatus

The EPM apparatus was employed according to the protocol described by Murade et al (2021) [11]. Rats who underwent chronic stress were alienated into five groups (*n = 6*); Group I: treated with vehicle; Group II: Chronic stress; Group III: Chronic stress +PG (100 mg/kg, p.o.); Group IV: Chronic stress +HT (50 mg/kg, p.o.); Group V: Chronic stress +HTN (50 mg/kg, p.o.) for 21 days, 1 hr before footshock experiment followed by the EPM experiment on the 22^nd^ day.

The rat was positioned on the maze’s central platform with its head to an open arm and subjected to recording their time spent in open arms and the number of open and closed arm entrants over 5 min. All four paws must be on the arm to define an entry. After each test, the maze’s platform was cleaned with 70% ethanol.

#### Statistical analysis

Statistical significance of EPM behavioral data was examined using one-way analysis of variance (ANOVA) followed by Bonferroni’s multiple comparisons test. At p<0.05, the results were considered significant and were stated as mean ± SEM.

## Results and discussion

### Preparation of HTN

Preliminary investigations demonstrated a significant influence of the process parameters, including drug to PC ratio (X_**1**_), reaction temperature (X_**2**_,°C) and the reaction time (X_**3**_, h) on entrapment efficiency, ranging from 68.19 to 97.65% (Table 2) [8].

Multiple correlation coefficient (R^2^) value of 0.9813 and low predicted residual error sum of square (PRESS) value of 118.00 confirmed that the quadratic model fits the data well. R^2^ value of 0.9813 corroborates that the quadratic model can predict the 98.13% variations in entrapment efficiency. As shown in ANOVA results (Table 3), a model F value (58.38; P<0.0001) depicts the statistical significance of the quadratic model. Three linear [X_1_, X_2_, and X_3_] and two quadratic [A² and B²] terms significantly affected EE. The predicted R^2^ (0.8756) was close to the adjusted R^2^ (0.9645). Adequate precision (29.29) was also found to be acceptable. Generally, a ratio greater than 4 is desirable.

**Table 3:** ANOVA of the quadratic model.

Source	Coeff.	Sum of Squares	Df	Mean Square	F-value	p-value
**Model**		931.19	9	103.47	58.38	< 0.0001^a^
A-Drug: PC ratio	+5.13	368.46	1	368.46	207.90	< 0.0001^a^
B-Reaction temperature	+4.50	283.22	1	283.22	159.81	< 0.0001^a^
C-Reaction time	+4.16	242.54	1	242.54	136.85	< 0.0001^a^
AB	-0.4363	1.52	1	1.52	0.8591	0.3758
AC	-0.2063	0.3403	1	0.3403	0.1920	0.6706
BC	-0.3763	1.13	1	1.13	0.6390	0.4426
A²	-0.7775	9.52	1	9.52	5.37	0.0429^a^
B²	-1.28	25.77	1	25.77	14.54	0.0034^a^
C²	+0.1308	0.2694	1	0.2694	0.1520	0.7048
**Residual**		17.72	10	1.77		
Lack of Fit		14.75	5	2.95	4.96	0.0517^b^
Pure Error		2.97	5	0.5946		

a: Significant

b: Non-significant.

Polynomial equations (full) relating the response yield (%) to the transformed factors can be given as Eq (4),

$${\text{Y}}_{1}=+86.07+5.13\text{A}+4.50\text{B}+4.16\text{C}-0.4363\text{AB}-0.2063\text{AC}-0.3763\text{BC}-0.7775{\text{A}}^{2}-1.28{\text{B}}^{2}+0.1308{\text{C}}^{2}$$ (4)

Factors showing non-significant (p>0.05) effects were excluded, and the reduced model can be written as Eq (5),

$${\text{Y}}_{1}=+86.07+5.13\text{A}+4.50\text{B}+4.16\text{C}-0.7775{\text{A}}^{2}-1.28{\text{B}}^{2}$$ (5)

Multiple linear regression analysis (Table 3) discovered the positive correlation of coefficients b_**1**_, b_**2**_, and b_**3**_. In other words, increasing X_**1**_ and X_**2**_, and X_**3**_ enhanced the entrapment of HT. F values of 207.90 confirmed that the drug: PC ratio had a prominent effect on EE (%). X_**1**_X_**2**_, X_**1**_X_**3**_ and X_**2**_X_**3**_ showed a negative but non-significant effect on %EE.

**Fig 1:**
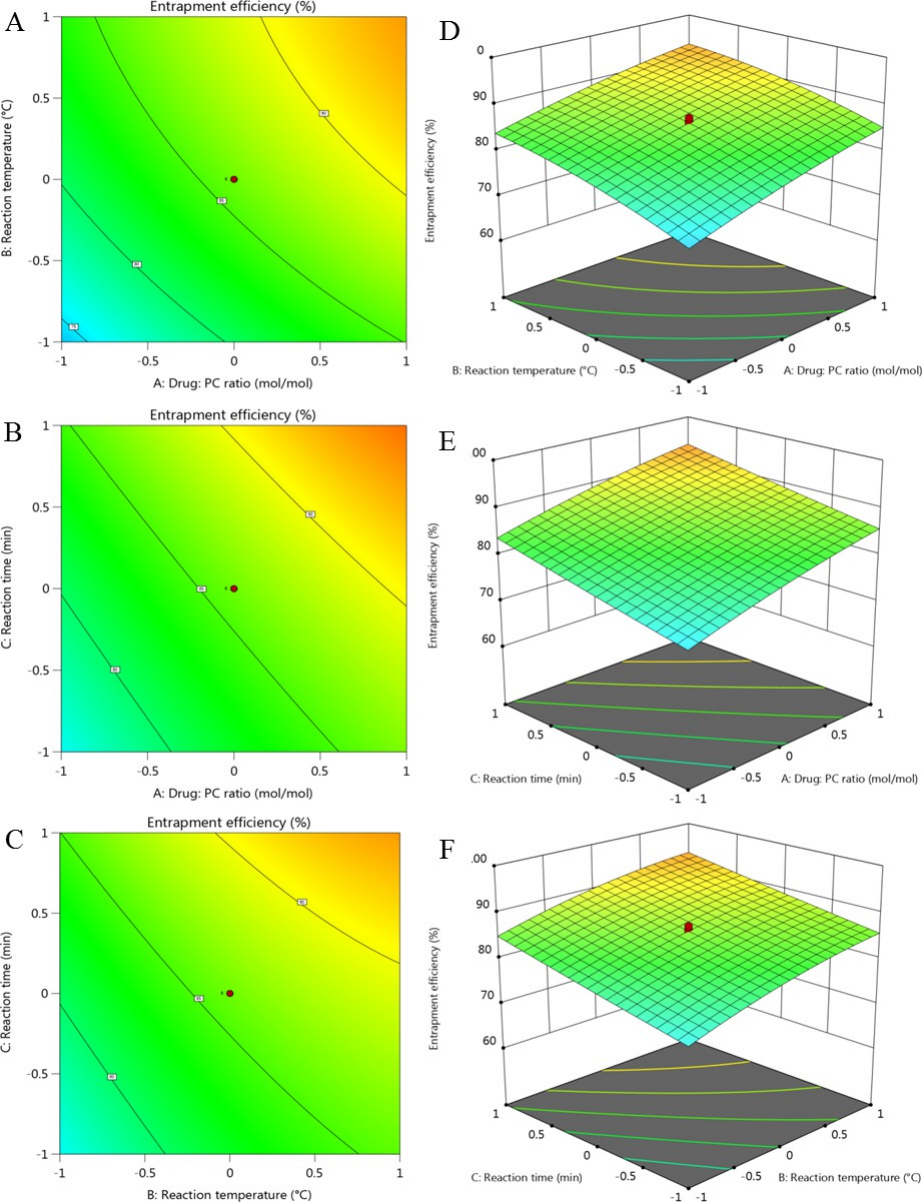
2D counter plots (A-C) and 3D surface plots (D-F) showing the effects of independent variables on EE (%).

Central composite design data derived from twenty batches were subjected to Design Expert® Version 13 (Stat-Ease, Inc., Minneapolis, MN) to generate interpolated values. Contour (Fig 1A–1C) and response surfaces plots (Fig 1D–1F) (based on the central composite design) illustrated the significant influence of X_1_, X_2_ and X_3_ on the EE. Moreover, higher EE was associated with increasing concentrations of X_1_, X_2_ and X_3_. As a result of these experimental findings and the multiple regression model, optimal values for the analyzed parameters were found as 1:2.89 (drug-to-phospholipid ratio), 56°C (reaction temperature), and reaction time (156 min).

Detailed model fitting data, model summary statistics, actual vs predicted plots and different model diagnostic plots were generated and are reported in the supplementary data (Supporting Information).

### Validation of the model

The model was validated by formulating an additional batch of HTN with optimal settings of the analyzed parameters, i.e., X_1_- 1: 2.89, X_2_- 56°C and X_3_- 156 min. Table 4 depicts the data of the HTNs’ predicted EE (96.92%) derived from the model and the actual EE (94.29%) attained through formulation, signifying the models’ feasibility and rationality. Furthermore, the bias (%) of less than 3% (2.71%) derived from Eq (6) confirms that the developed model is relatively robust [8].


$$\text{Bias}\left(\%\right)=\text{Predicted}\;\text{value}-\text{Observed}\;\text{Value}/\text{Predicted}\;\text{value}\;\text{X}\;100$$ (6)

**Table 4:** Comparison of the observed and predicted values of HTN prepared under predicted optimum conditions.

Response Variable	Predicted Value	Observed value*	Bias (%)
Entrapment efficiency (%)	96.92	94.29 ± 1.24	2.71

* Values represent mean ± standard deviation (n = 3).

### Entrapment efficiency

The present study revealed good encapsulation efficiency, i.e., 97.65% % (w/w) as estimated by UV spectrophotometry.

### Fourier transform—infrared spectroscopy (FT-IR)

**Fig 2:**
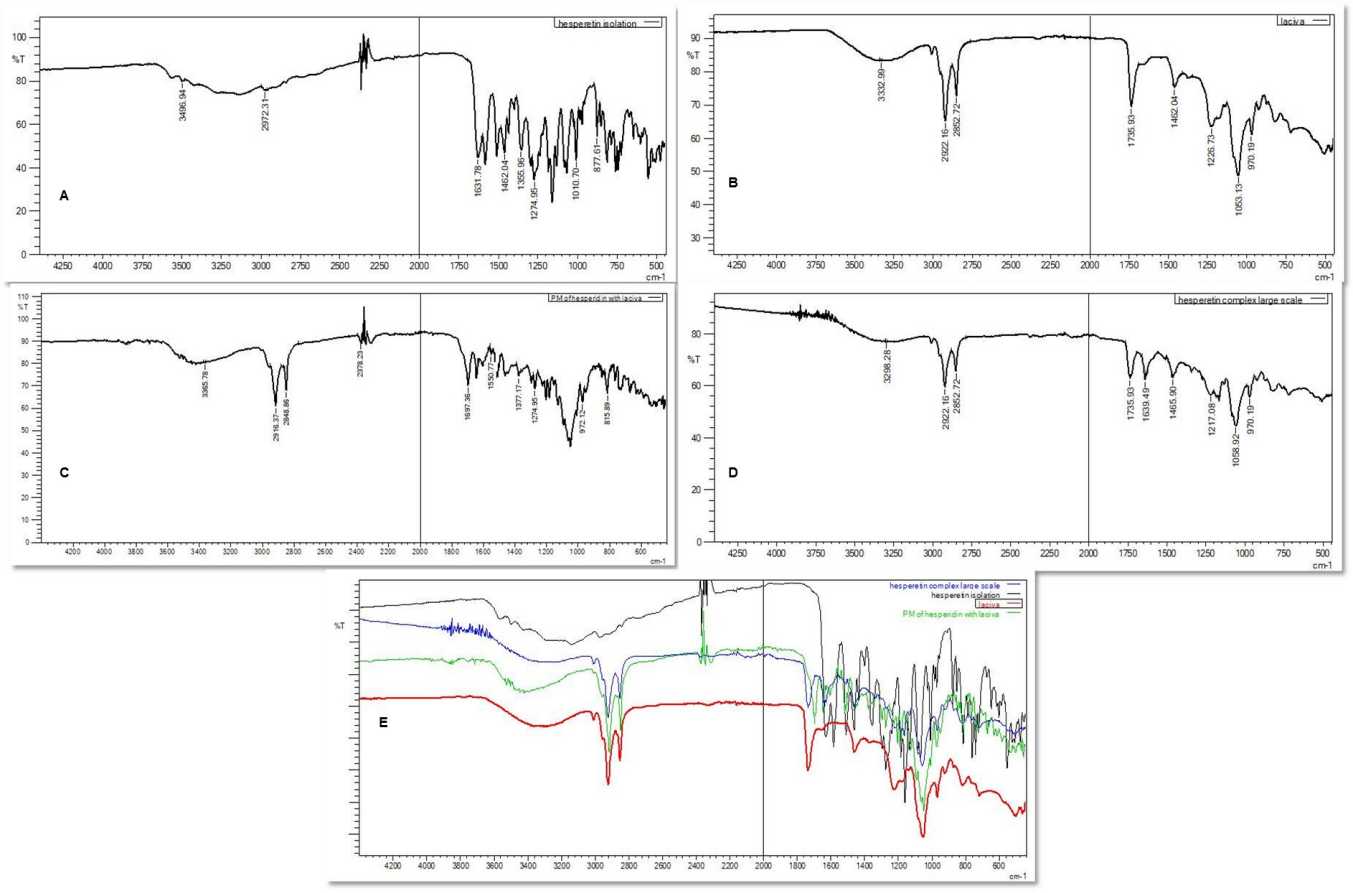
FT-IR spectra of HT (A), PC (B), PM (C), HTN (D), and overlay spectrum (E).

The FT-IR analysis (Fig 2) confirmed the complex formation between the HT and PC. The FT-IR spectrum of HT (Fig 2A) demonstrated a peak at 3496.94 cm^-1^ due to the phenolic OH stretching vibration (hydroxyl group). The peak at 2942.31 cm^-1^ was due to the alkyl C-H stretch present in the extract. The peak at 1631.78 cm^-1^ was contributed by C = O stretching (carboxyl group), whereas the peak at 1462.04 cm^-1^ was due to C = C aromatic bending. The PC spectrum (Fig 2B) exhibited a peak at 2922.16 cm^-1^ due to the C-H stretching of a long fatty acid chain. The P = O (phosphomoyl) group and ester linkage confirmed the peaks at 1735.93 cm^-1^, and 1226.73 cm^-1^, respectively. P-O-C and N(CH_3_)_3_ stretching bands were seen at 1053.13 cm^-1^, and 970 cm^-1^, respectively [8,12]. These characteristic peaks still exist in the FT-IR spectrum of PM (Fig 2C) and HTN (Fig 2D), suggesting no interaction between HT and PC.

In the HTN complex (Fig 2D and 2E), the absorption peak of hydroxyl (O–H) showed a broad peak with stretching of HT shifted to a lower wavenumber, signifying the strong hydrogen bonding between the PC and HT hydroxyl groups in the naturosomal form. The HTN spectrum showed no changes in the PC’s two long aliphatic chain fatty acid bands, indicating their non-involvement in the HTN formation. A shift in PC’s P = O absorption band to a higher wavenumber and P-O-C stretching vibrations to a lower wavenumber confirms the formation of HTN [13,14].

### Differential scanning calorimetry (DSC)

**Fig 3:**
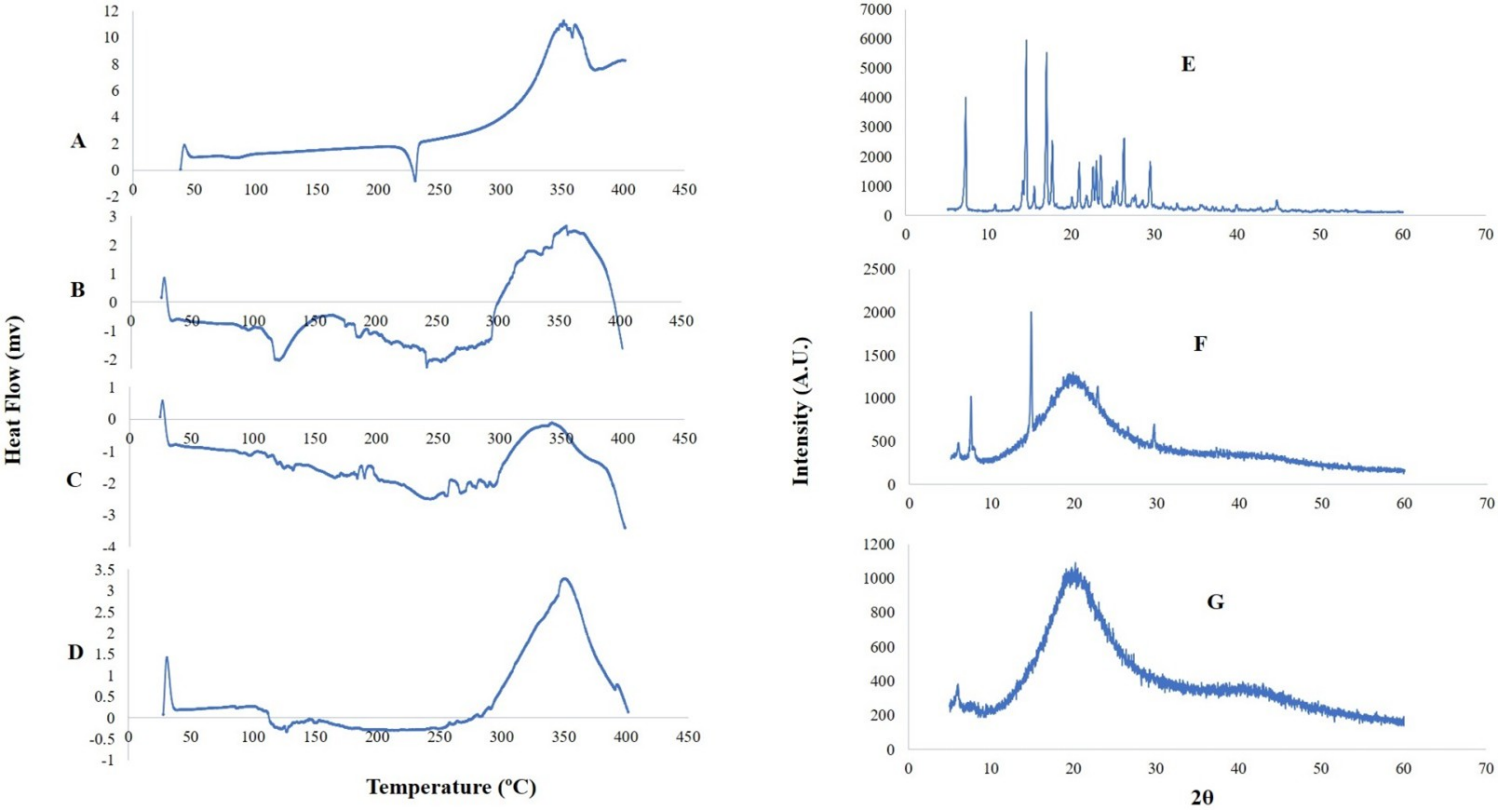
DSC thermogram of HT, PC, PM and HTN.

DCS thermograms of HT, PC, and PM are depicted in Fig 2. HT (Fig 3A) exhibited an endothermic peak at 230.4°C. PC (Fig 3B) demonstrated two major endothermal peaks: the first peak at 119.33°C, perhaps owing to the heated movement of phospholipids’ polar head group, and the sharp second peak at 242.05°C due to the melting of a carbon-hydrogen chain of phospholipids during gel to liquid crystalline phase transition [16]. The PM (Fig 3C) exhibits two endothermal peaks at 112.27°C and 249.95°C.

HTN thermogram (Fig 3D) displayed peaks at 110.53°C and 251.0°C, which differed from the peaks of HT and PC. It is possible that a lower melting point and enthalpy are responsible for the HT’s amplified solubility and reduced crystallinity. The disappearance of the original peaks of HT and PC from the HTN thermogram and a lower phase transition temperature than PC confirmed the formation of the drug-phospholipid complex [8].

### X-ray diffraction analysis (XRD)

It is common to use X-RPD to determine the crystalline nature. The crystallinity of the HT (Fig 3E) was clearly visible in its diffraction peaks. From the diffractogram of HTN (Fig 3F), it was clear that most drug-associated crystalline peaks had vanished, confirming the formation of a drug-phospholipid complex [12].

### Photomicroscopy

**Fig 4:**
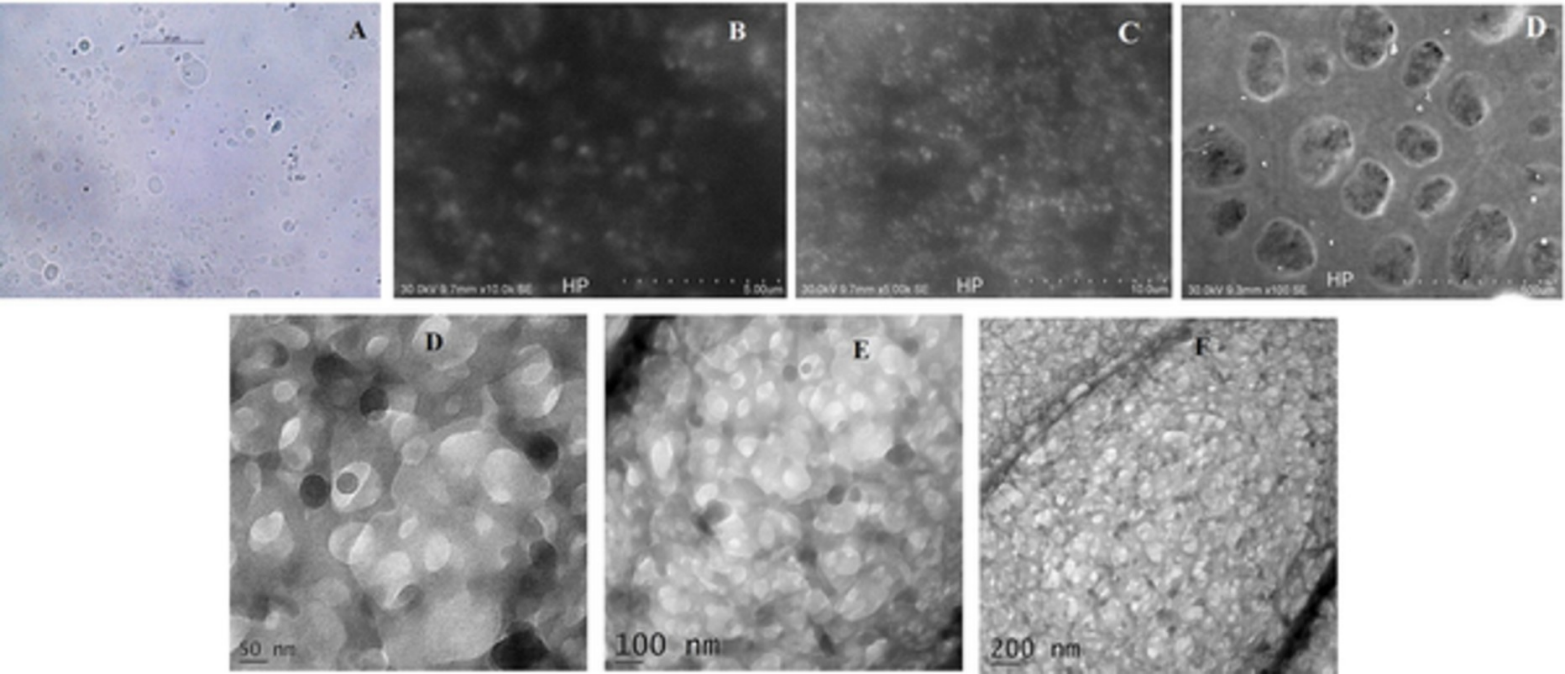
Photomicroscopic images of HTN (A), SEM images of HTN (B and C), and TEM images of HTN (D and E).

Fig 4A shows a microscopic view of the complex, revealing spherical structures’ presence. The drug was intercalated into PC’s lipid layers to create vesicle-like structures. The drug particles’ surface morphology showed that they were linked to the phospholipid and formed complexes of varying sizes [8].

### Scanning electron microscopy (SEM)

An SEM image (Fig 4B and 4C) of the HTN demonstrated an irregularly shaped HTN with rough surface morphology. HT was physically entrapped by PC and entirely converted into a phospholipid complex, imparting an amorphous nature and losing its crystallinity [16].

### Transmission electron microscopy (TEM)

TEM analysis of HTN (Fig 4D and 4E) reveals the spherical-shaped vesicular structure [17].

### Particle size and zeta potential analysis

**Fig 5:**
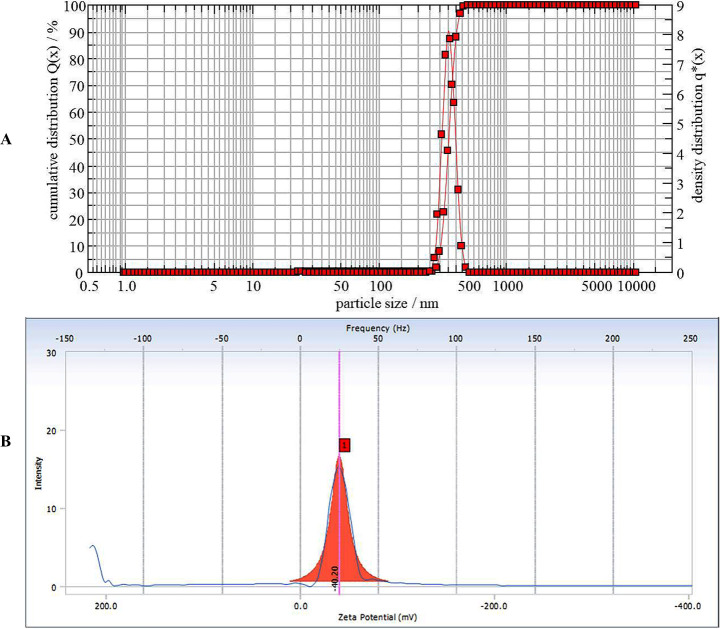
Particle size (A) and Zeta Potential (B) analysis of HTN.

The HTN has a mean particle size of 350.11 nm (Fig 5A). Most particles’ surface area/volume (SA/V) ratio is inversely proportional to particle size. As a result, the entrapped drug is more easily released from the phospholipid-drug complex when smaller particles have higher SA/V. The lymphatic system takes up larger particles (5 mm), while endocytosis allows smaller particles (500 nm) to cross the epithelial cell membrane [16,18,19].

The naturosomal stability can also be ascertained by their zeta potential measurement. Physical stability is indicated by zeta potential values greater than -30 mV, which is considered acceptable [20]. The zeta potential of the HTN was -40.20 mV (Fig 5B).

### Solubility studies

The results of the solubility analysis are represented in Table 5.

**Table 5:** Solubility studies of HT, PC, and HTN.

SOLVENT	HT	PC	COMPLEX
Ethanol	Soluble	Soluble	Soluble
DCM	Soluble	Soluble	Soluble
Chloroform	Sparingly Soluble	Soluble	Soluble
DMSO	Soluble	Insoluble	Insoluble
Hexane	Insoluble	Soluble	Soluble
Water	Insoluble	Turbid solution	Micellar solution

### Apparent solubility studies

Poor aqueous solubility and comparatively higher solubility in n-octanol specify the lipophilic nature of the HT. Conversely, the low solubility of the drug is imparted by its crystalline and highly lipophilic nature. Significant (p<0.05) difference was observed between the aqueous and n-octanol solubility of HT and NTN. Reduced molecular crystallinity and the amphiphilic nature of the HTN might have contributed to a substantial upsurge in the solubility by 10 folds in the aqueous medium compared to a 1.27 folds increment in n-octanol (Table 6) [15]. This could also be assigned to hydrogen bond formation and electrostatic interaction between HT and phospholipid molecules [21]. The–OH groups of the phenol rings of HT might have been involved in hydrogen bonding, whereas the aromatic rings could have been associated with hydrophobic interaction [22].

**Table 6:** Apparent solubility studies of HT, PM, and HTN.

Test drug	Aqueous solubility(μg/mL)	n-octanol solubility(μg/mL)
HT	56.72	692.8
PM	72.13	701.2
HTN	564.54	882.1

### Dissolution study

**Fig 6:**
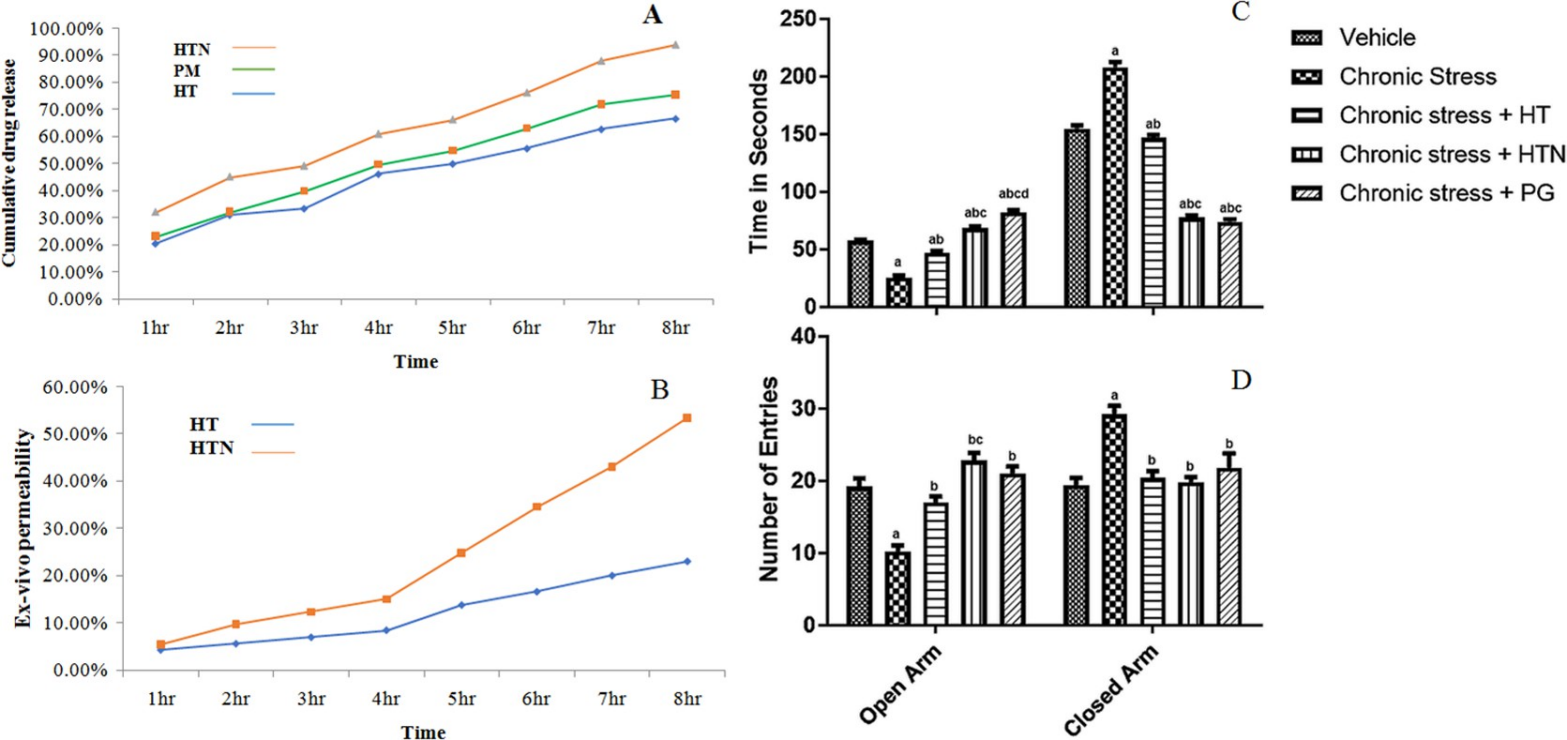
*In-vitro* dissolution study of HT, PN, and HTN (A), *Ex-vivo* permeability of HT and HTN, and *in-vivo* activity of HTN (C and D). Values are mean ±S.E.M. (n = 5). Where a: P < 0.05 vs. Vehicle, b: P < 0.05 vs. Chronic stress, c: P < 0.05 vs. Chronic stress + HT and d: P < 0.05 vs. Chronic stress + HTN. One-way ANOVA, followed by post hoc Bonferroni’s multiple comparison test.

The dissolution studies (Fig 6A) showed 66.74% release of HT, which was not significantly different (p<0.05) from PM, i.e., 75.62%. However, HTN showed the highest and most significant (p<0.05) dissolution rate (93.94%) compared to HT and PM, confirming a positive effect of amphiphilic phospholipid molecules on drug dissolution [23]. The dissolution rate is a function of wettability, crystal structure, and crystal size. Enhanced solubility, nanosizing, high surface area, and considerable amorphous state of HT in the generated naturosomes could all contribute to the greater HT dissolution rate from the HTN [24].

### *Ex-vivo* permeability study

Fig 6B depicts an *ex-vivo* permeability analysis of HT and HTN. At the end of 8 h, 23.1% of the HT had permeated through the rat skin, whereas HTN had 53.42%. The results differed significantly at p<0.05 [18].

### *In-vivo* activity

On the 22^nd^ day in EPM, rats treated with test drugs were assessed for anxiety and adaptogenic effects. A one-way ANOVA discovered that treatments had a statistically significant (p<0.0001) effect on the time spent in open and closed arms and the total entrants in EPM. Moreover, a significant upsurge in anxiety-related indices and a significant decline in time spent (p<0.05) and open arm entries (p<0.01) than the vehicle-treated animals were observed. Furthermore, compared to the chronic stress-treated group, PG and HTN administration significantly (p<0.001) altered time spent and open arm entries. HT enhanced the open arm entries (p<0.05) but not the time spent (p>0.05). While PG, HT, and HTN significantly (p<0.01, p<0.05, and p<0.01, respectively) decreased time spent and closed arm entries than chronic stress animals (Fig 6C and 6D). As compared to HT, HTN treatment resulted in significantly (p<0.05) more time spent in the open arm and less time spent in the closed arm, suggesting the superior therapeutic efficacy of HTN [11].

## Conclusion

In the current investigation, HT entrapped naturosomes for enhanced delivery were successfully fabricated and optimized using a central composite design. The QbD approach with different rational combinations of formulation variables provided optimal settings for preparing naturosomes. The aqueous solubility of HT is the major constraint that limits its therapeutic applicability. Herein, entrapment of HT in naturosomes drastically enhanced its aqueous solubility and dissolution rate, thus could amplify its overall therapeutic effectiveness. In addition, high permeability of HTN across the rat’s skin than pure HT supported the above findings. Further, *in-vivo* testing based on footshock induced stress and EPM model revealed significant activity of HTN than HT, confirming the reliability of *in-vitro* findings. Conclusively, naturosomes could improve drug solubility, efficacy, and colloidal stability.

## Supporting information

S1 File Supporting information. (PDF)

S1 Graphical abstract (TIF)
