# Supplementary material for: Preparation, characterization, and evaluation (in-vitro, ex-vivo, and in-vivo) of naturosomal nanocarriers for enhanced delivery and therapeutic efficacy of hesperetin
Source: PLoS One. 2022 Nov 3;17(11):e0274916. doi: 10.1371/journal.pone.0274916 (PMC9632909; doi:10.1371/journal.pone.0274916)
Supplement: S1 File — (PDF) [file pone.0274916.s001.pdf]

Supporting Information

Preparation, characterization, and evaluation (*in-vitro, ex-vivo,* and *in-vivo*) of naturosomal nanocarriers for enhanced delivery and therapeutic efficacy of hesperetin

Shailendra Gurav[1*], Poonam Usapkar[1], Nilambari Gurav[2], Sameer Nadaf [3], Muniappan Ayyanar[4], Rucheera Verekar[1], Ritesh Bhole[5], Chintha. Venkataramaiah[6], Goutam Jena[7], Rupesh Chikhale[8*]

[1] Department of Pharmacognosy, Goa College of Pharmacy, Panaji, Goa University, Goa, India

[2] PES's Rajaram and Tarabai Bandekar College of Pharmacy, Ponda, Goa University, Goa-India

[3] Sant Gajanan Maharaj College of Pharmacy, Mahagaon, Chinchewadi, Maharashtra, India

[4] Department of Botany, A. Veeriya Vandayar Memorial Sri Pushpam College (Autonomous), Affiliated to Bharathidasan University, Poondi, Thanjavur, Tamil Nadu, India

[5] Dr. D. Y. Patil Institute of Pharmaceutical Sciences and Research, Pimpri, Pune, Maharashtra, India

[6] Department of Medical Environmental Biology and Tropical Medicine, School of Medicine, Kangwon National University, Republic of Korea

[7]Roland Institute of Pharmaceutical Sciences, Berhampur, Odisha, India

[8] UCL School of Pharmacy, 29−39 Brunswick Square, London WC1N 1AX, United Kingdom
*Corresponding authors:

r.chikhale@ucl.ac.uk (RC); shailendra.gurav@nic.in (SG)

Data from the experiment were fitted to quadratic, two-factor interaction, and linear models. The best fit model was chosen based on R^2 and the Prediction Residual Sum of Squares (PRESS) value. Additionally, the greatest Adjusted R^2 and the Predicted R^2 were used to choose the model. Table S1 and Table S2 depict specific findings. The model is likely significant given the Model F-value of 58.38. An F-value this large could only happen owing to noise in 0.01% of cases. The Lack of Fit F-value of 4.96 indicates a 5.17 percent possibility that noise could cause a Lack of Fit F-value.

The quadratic model's R^2 value of 0.9813 confirms its ability to estimate variations in entrapment efficiency of 98.13% (Table S3). The Predicted R^2 of 0.8756 and the Adjusted R^2 of 0.9645 are reasonably in agreement; the difference is less than 0.2. Adeq Precision measures the ratio of signal to noise. A ratio of at least 4 is preferred. An adequate signal is indicated by a ratio of 29.299. This model can therefore be used to explore the design space.

Table S1: Model fitting data

**Source**	**Sequential p-value**	**Lack of Fit p-value**	**Adjusted R^2**	**Predicted R^2**	
Linear	< 0.0001	0.0167	0.9316	0.9043	
2FI	0.8591	0.0101	0.9204	0.8406	
**Quadratic**	**0.0108**	**0.0517**	**0.9645**	**0.8756**	**Suggested**
Cubic	0.0324	0.2986	0.9874	0.8166	**Aliased**

Table S2: Model Summary Statistics

**Source**	**Std. Dev.**	**R^2**	**Adjusted R^2**	**Predicted R^2**	**PRESS**	
Linear	1.85	0.9424	0.9316	0.9043	90.77	
2FI	1.99	0.9455	0.9204	0.8406	151.28	
**Quadratic**	**1.33**	**0.9813**	**0.9645**	**0.8756**	**118.00**	**Suggested**
Cubic	0.7930	0.9960	0.9874	0.8166	174.06	Aliased

Table S3: Fit Statistics of Quadratic Model

**Std. Dev.**	1.33	**R^2**	**R^2**	0.9813
**Mean**	84.72	**Adjusted R^2**	**Adjusted R^2**	0.9645
**C.V. %**	1.57	**Predicted R^2**	**Predicted R^2**	0.8756
		**Adeq Precision**	**Adeq Precision**	29.2990

When all other factors are maintained constant, the coefficient estimate shows the expected change in output per unit change in factor value. The average response of all the runs is the intercept in an orthogonal design. Based on the factor settings, the coefficients modify the average around it. When the factors are orthogonal, the VIFs are 1. When the factors are multi-colinear, the VIFs are greater than 1. The higher the VIF, the more severe the correlation of the factors. VIFs under 10 are generally considered tolerable. Table S4 presents comprehensive findings.

Table S4: Coefficients in Terms of Coded Factors

**Factor**	**Coefficient Estimate**	**df**	**Standard Error**	**95% CI Low**	**95% CI High**	**VIF**
Intercept	86.07	1	0.5435	84.86	87.28	
A-Drug: PC ratio	5.13	1	0.3558	4.34	5.92	1.0000
B-Reaction temperature	4.50	1	0.3558	3.71	5.29	1.0000
C-Reaction time	4.16	1	0.3558	3.37	4.96	1.0000
AB	-0.4363	1	0.4707	-1.48	0.6125	1.0000
AC	-0.2063	1	0.4707	-1.25	0.8425	1.0000
BC	-0.3763	1	0.4707	-1.42	0.6725	1.0000
A^2	-0.7775	1	0.3354	-1.52	-0.0301	1.03
B^2	-1.28	1	0.3354	-2.03	-0.5318	1.03
C^2	0.1308	1	0.3354	-0.6166	0.8782	1.03

The importance of the proposed models could be shown by the plot of predicted versus actual EE (%) (Fig. S1 and Table S5). The impact of a single independent variable on a response is shown by a perturbation plot (Fig. S2), while other variables are kept constant at a reference point. The curve's greatest curvature defined the dependent variable's sensitivity to the independent variables. This demonstrates that the drug: PC ratio and reaction temperature significantly impact the EE%. The higher F values of 207.90 and 159.81, respectively, further supported this.

Table S4: Actual and predicted values of %EE

Run Order	Actual Value	Predicted Value	Residual	Leverage	Internally Studentized Residuals	Externally Studentized Residuals	Cook's Distance	Influence on Fitted Value DFFITS	Standard Order
1	79.58	80.88	-1.30	0.661	-1.679	-1.880	0.549	-2.623[(1)]	2
2	68.19	69.34	-1.15	0.661	-1.479	-1.587	0.426	-2.215[(1)]	1
3	86.12	86.07	0.0483	0.167	0.040	0.038	0.000	0.017	16
4	75.53	74.44	1.09	0.619	1.322	1.381	0.284	1.760	11
5	86.39	86.07	0.3183	0.167	0.262	0.249	0.001	0.112	15
6	88.56	89.76	-1.20	0.661	-1.545	-1.679	0.465	-2.343[(1)]	4
7	93.77	92.62	1.15	0.619	1.394	1.473	0.316	1.878	10
8	86.03	86.07	-0.0417	0.167	-0.034	-0.033	0.000	-0.015	19
9	89.49	90.02	-0.5345	0.619	-0.650	-0.631	0.069	-0.804	12
10	74.26	74.85	-0.5933	0.619	-0.722	-0.704	0.085	-0.897	9
11	97.65	96.92	0.7328	0.661	0.945	0.939	0.174	1.311	8
12	85.95	86.07	-0.1217	0.167	-0.100	-0.095	0.000	-0.042	17
13	92.41	93.67	-1.26	0.619	-1.537	-1.669	0.384	-2.128[(1)]	14
14	88.98	89.55	-0.5666	0.661	-0.731	-0.712	0.104	-0.994	6
15	79.61	78.83	0.7838	0.661	1.011	1.012	0.199	1.412	5
16	80.11	79.96	0.1526	0.661	0.197	0.187	0.008	0.261	3
17	84.78	86.07	-1.29	0.167	-1.063	-1.071	0.023	-0.479	20
18	88.83	87.94	0.8881	0.661	1.145	1.166	0.255	1.627	7
19	87.16	86.07	1.09	0.167	0.896	0.886	0.016	0.396	18
20	81.07	79.25	1.82	0.619	2.209	2.929	0.793	3.734[(1)]	13

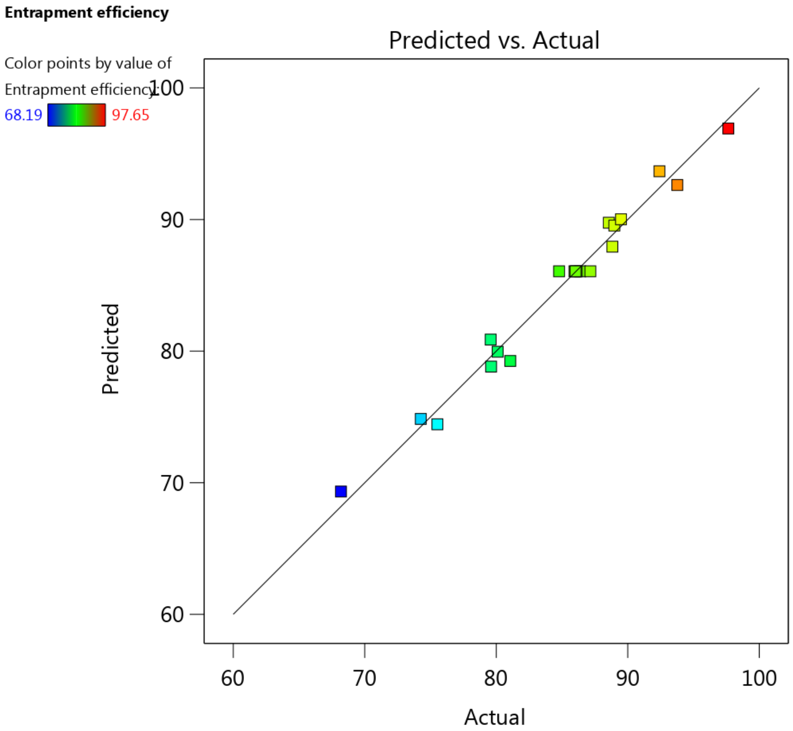

Figure S1: Plot of predicted Vs, actual values of EE (%)

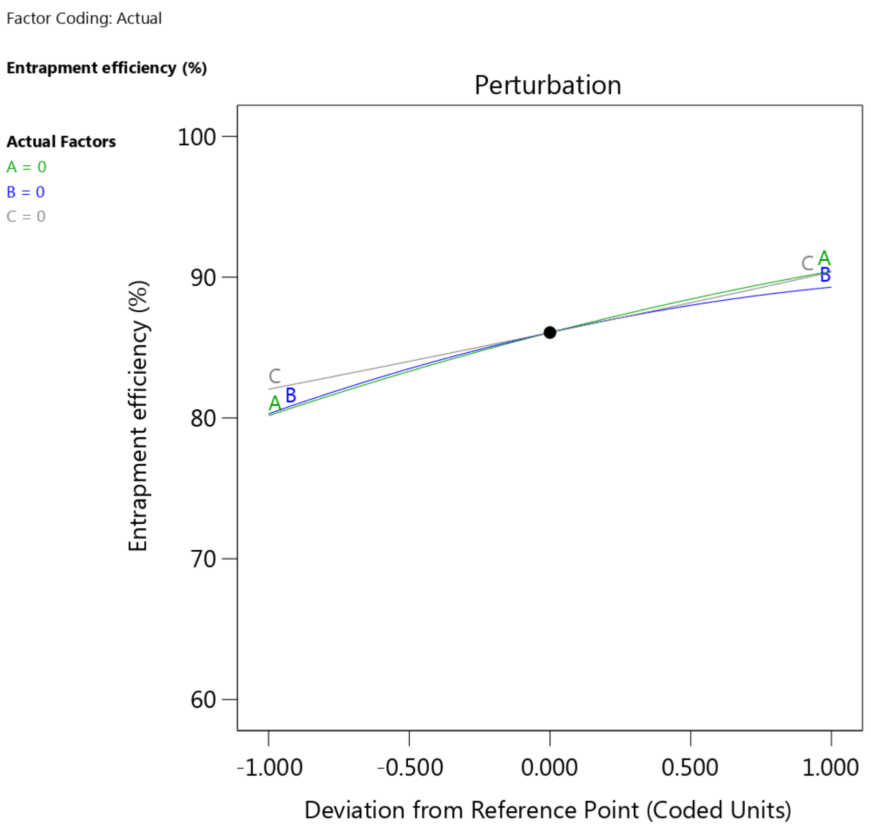

Figure S2: Perturbation plot depicting the effect of variables on EE (%)

Plots for model diagnosis were created, as seen in Figures S3–S7. The quality of the regression can be evaluated using the normal residual plot. The residuals' normal probability plot is roughly linear, confirming the assumption that the error terms are normally distributed (Fig. S3). The random distribution that created a nearly horizontal zone around the residual line was also displayed in the Residual vs. Predictor Plot. From the common random distribution of the other residuals, no data points stood out (Fig. S4). A visualization of the residuals against the experimental run order is known as a residual vs. run plot. It searches for hidden factors that might have affected the experiment's response.

The plot displayed a random scattering, as seen in Fig. S5. Cook's distance demonstrating the scaled change in fitted values was also calculated. Cook's distance summarizes how much a

regression model changes when the ith observation is removed. This is useful for identifying outliers in the *X* values. Cook's distance shows the influence of each observation on the fitted response values. An observation with Cook's distance larger than three times the mean Cook's distance might be an outlier. A regression model's Cook's Distance measures how much it changes when the ith observation is taken out of the equation. This can be used to spot outliers in the X values. Cook's distance illustrates how each observation affects the values of the fitted response. A measurement with a Cook's distance more than three times the average could be an outlier. It was determined that Cook's distance was less than 1 (Fig. S6 and Table S4). Therefore, no point was deemed to be influential. Box-Cox transformation plot (Fig. S7) showed a λ value of 1. As a result, the original data were analyzed without any modifications.

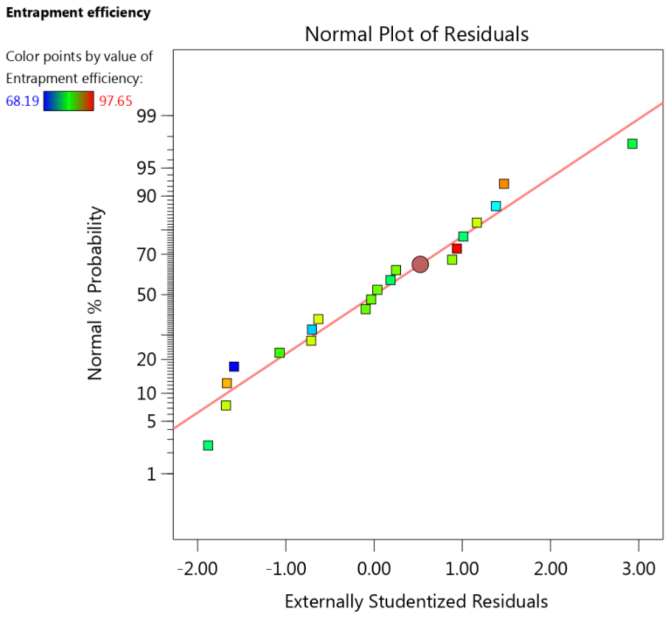

Figure S3: Normal plot of residuals

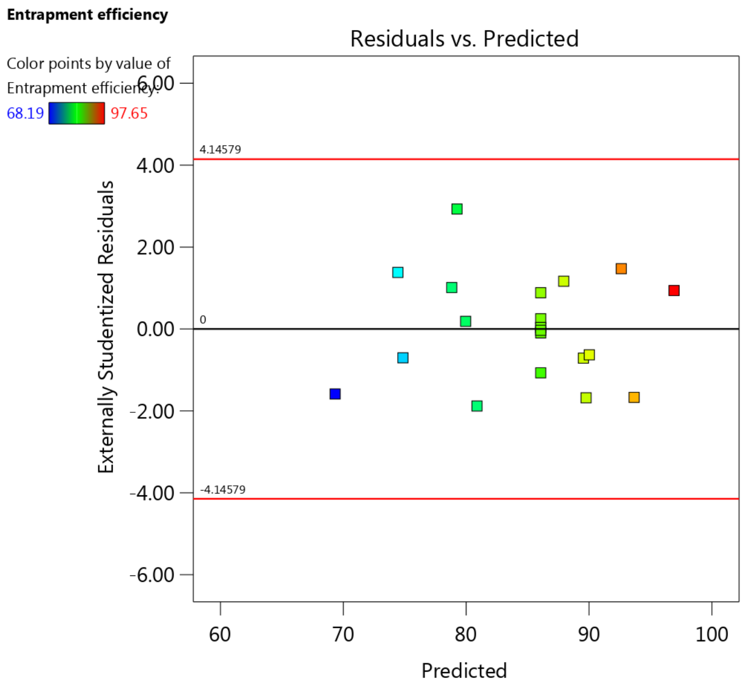

Figure S4: Residuals vs. predicted plot

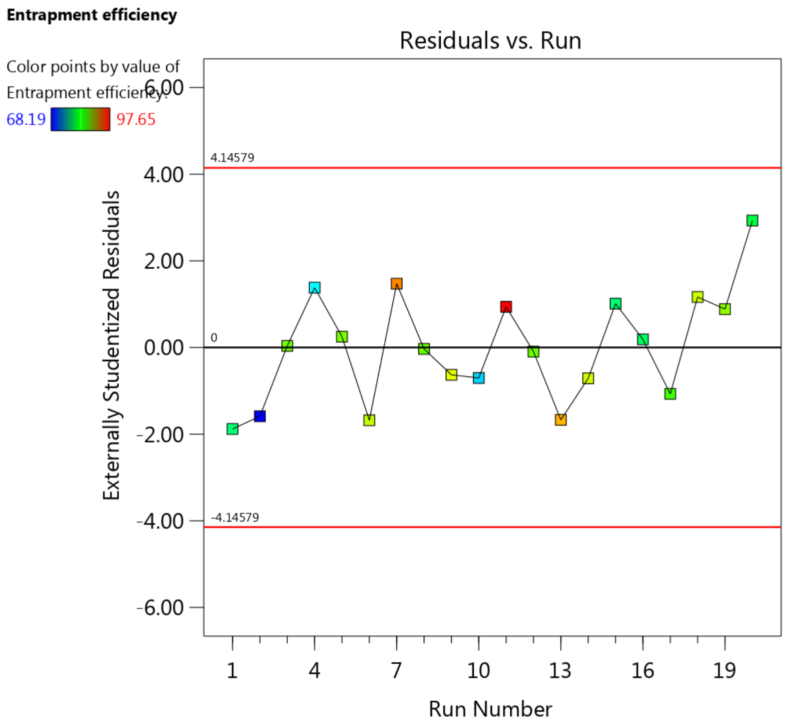

Figure S5: Residual vs. Run plot

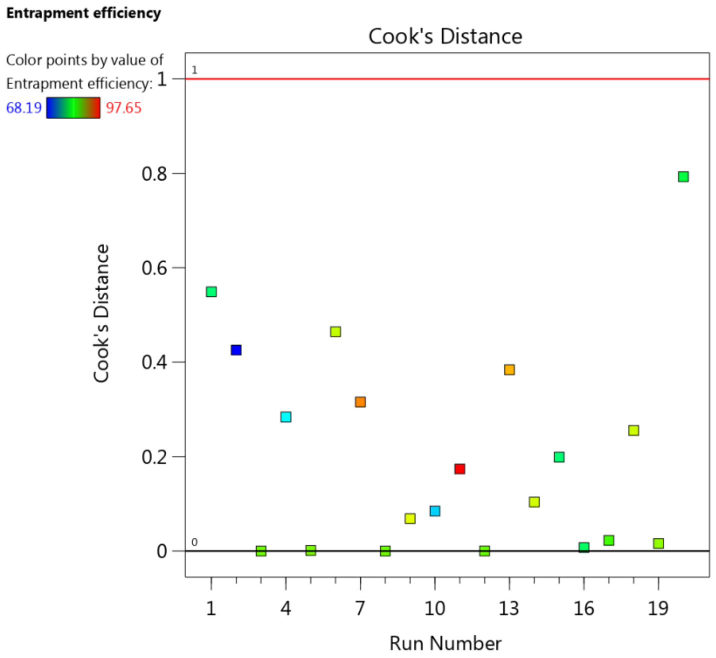

Figure S6: Identifying Outliers in Linear Regression with Cook distance

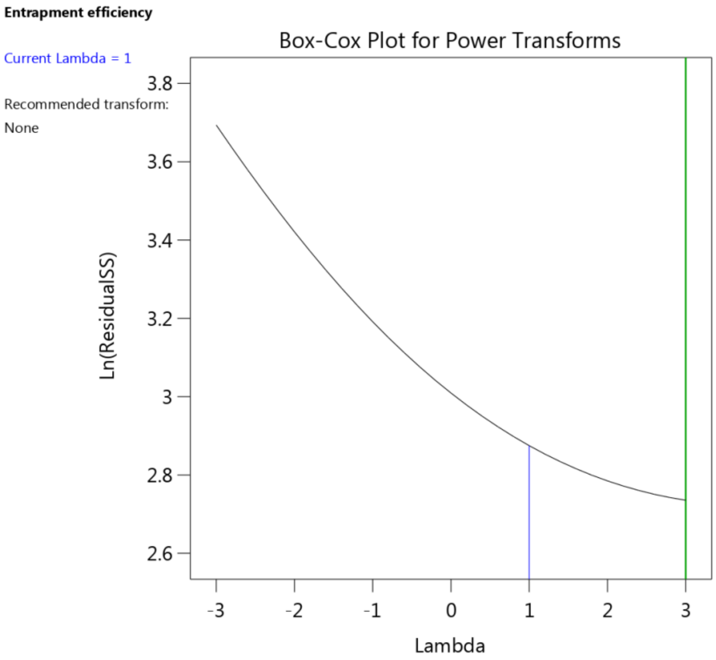

Figure S7: Box-Cox plot for power transforms
