# Supplementary figures and images for: Preparation, characterization, and evaluation (in-vitro, ex-vivo, and in-vivo) of naturosomal nanocarriers for enhanced delivery and therapeutic efficacy of hesperetin
Source: PLoS One. 2022 Nov 3;17(11):e0274916. doi: 10.1371/journal.pone.0274916 (PMC9632909; doi:10.1371/journal.pone.0274916)

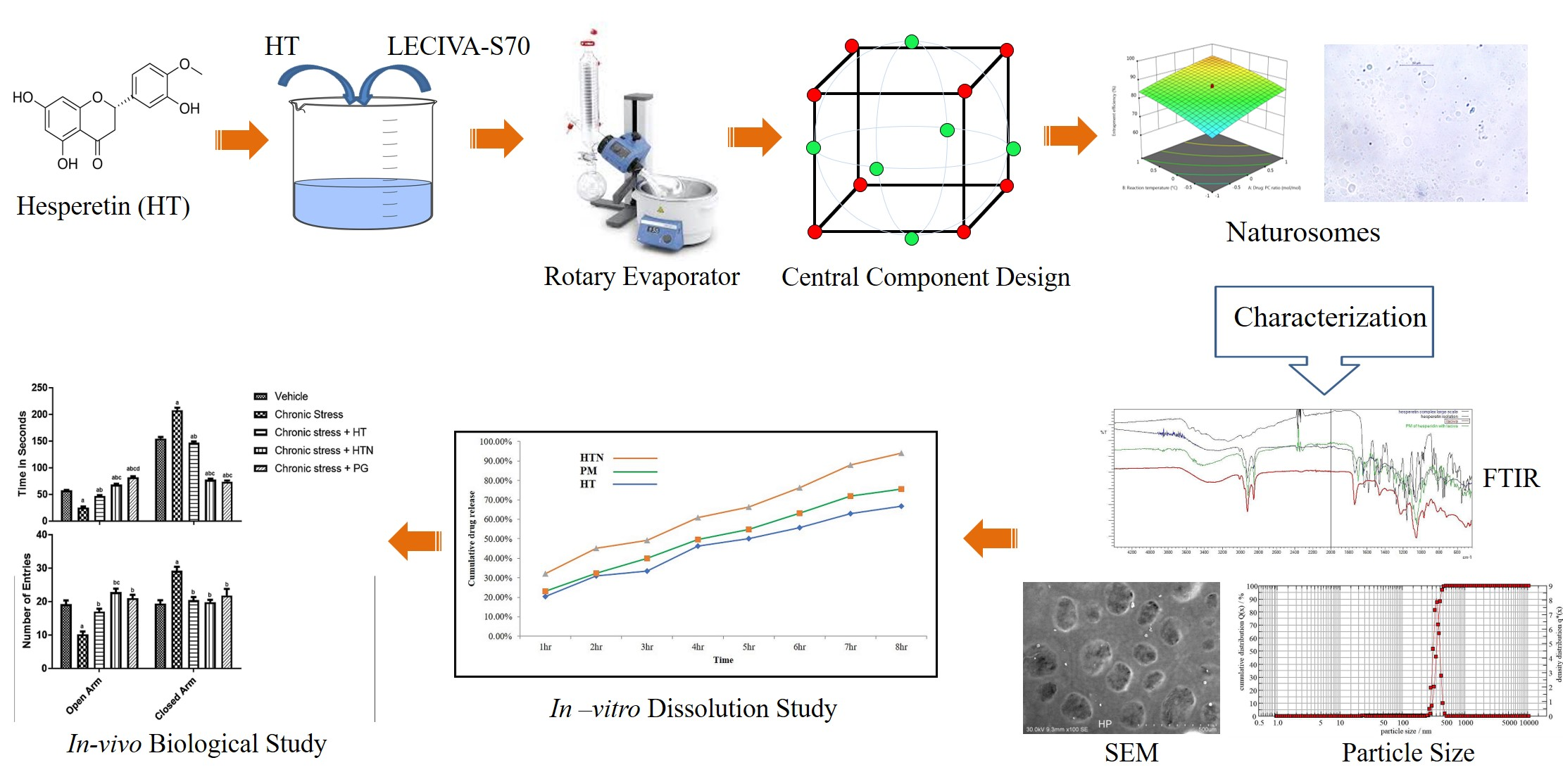

Supplement: S1 Graphical abstract — (TIF) [file pone.0274916.s002.tif]
